# Post-traumatic stress disorder in patients treated for schizophrenia: A cross-sectional study in the psychiatric department of Oujda, Morocco

**DOI:** 10.1016/j.amsu.2022.103651

**Published:** 2022-04-25

**Authors:** Salah-Eddine El Jabiry, Mohamed Barrimi, Bouchra Oneib, Fatima El ghazouani

**Affiliations:** a Department of Psychiatry, Mohammed VI University Hospital of Oujda, Morocco; b Faculty of Medicine and Pharmacy, Mohammed the First University of Oujda, Morocco

**Keywords:** Schizophrenia, Post-traumatic stress disorder, Stressful events, Cross sectional study

## Abstract

The prevalence of post-traumatic stress disorder (PTSD) in the general population is unavoidable and it seems that people who are suffering from severe psychiatric disorders especially schizophrenia, are more vulnerable to traumatic exposure and consequently to post traumatic stress disorder. The present work aims at determining the prevalence and the characteristics of the association between schizophrenia and PTSD since it isn't well known in Morocco.

**Materials and methods:**

We conducted a descriptive and analytical cross-sectional study over a period of three months from October 2019 to December 2019 at the Department of Mental Health and Psychiatric Diseases of the University Hospital Center Mohammed VI of Oujda.

**Results:**

The number of patients included in our study was 187 and the majority of them were male with a percentage of 76%. Several variables were evaluated. The prevalence of PTSD in the patients included in our study is 14%. In addition, the statistically significant variables were the presence of a stressful event (p = 0,001), the positive schizophrenia symptom score (PANSS P) (p = 0,031), the negative schizophrenia symptom score (PANSS N) (p = 0,005), the general schizophrenia symptom score (PANSS G) (p = 0,021), suicide risk (p < 0,001), and the presence of depression (p = 0,004).

**Conclusion:**

The comorbidity schizophrenia-PTSD exists with non-negligible prevalence. The risks of non-diagnosis of this comorbidity could lead to inappropriate treatments, a multiplication of care with no notable clinical improvement, poor therapeutic compliance and the alteration in the patients’ quality of life.

## Highlights

• The prevalence of this comorbidity in our study is 14%.
• Several factors were statistically significant with this association.

## 1 Introduction

Post-traumatic stress disorder (PTSD) is a pathology that may occur after direct exposure (Personal contact), or indirect exposure (witnessing or being informed by news) to an event or an incident in the context of death, threat of death, serious injury, or sexual assault, causing psychological trauma. It is characterized by the development of specific and long-lasting symptoms such as the repetition syndrome (reliving), the avoidance of stimuli evocative of the event, and neuro-vegetative hyper activation [1].

The prevalence of PTSD in the general population appears to be non-negligible. According to the ESEMED study (European Study of the Epidemiology of Mental Disorders) conducted in 2004, PTSD affects 2.9% of women and 0.9% of men [2]. However, this prevalence becomes more fundamental in a population of patients being treated against psychiatric disorders [[3], [4], [5]].

PTSD can exist on its own or as a part of certain psychiatric comorbidities, namely depressive disorder and substance use disorder such as alcohol or various drugs [6].

The association of schizophrenia with PTSD seems interesting. A review of the literature conducted in 2011, led towards an average of 12.4% [3]. Another review done in 2016 found significant percentages ranging from 0 to 57% PTSD in patients suffering from schizophrenia or schizophrenia spectrum disorder [7].

Some authors have observed an increase in positive and negative symptoms of schizophrenia when PTSD is associated [[10], [11], [12]]. Others have noted an increase in depressive symptoms [13,14]. PTSD is considered to be a very important risk factor for suicide; which can be the same when there is an association with a schizophrenic disorder [15]. These patients’ quality of life could be changed through the high use of somatic care services with frequent hospitalizations in psychiatry [16].

The purpose of this work is to shed the light on this comorbidity in our patients who have been treated for schizophrenia by studying the main prevalence and the characteristics of this association with an attempt to improve the management and the prognosis of several patients.

## 2 Patients and method

We conducted a descriptive and analytical cross-sectional study spread over a period of three months from October 2019 to December 2019.

In this study, we included patients stabilized at least partially, not disorganized, followed for schizophrenia within the Department of Mental Health and Psychiatric Diseases of the University Hospital Center Mohammed VI, of Oujda.

Non-consenting and unstable subjects in addition to the patients with the disability to answer the hetero questionnaire correctly were excluded from this work.

For each patient, information was collected using the hetero-questionnaire including socio-demographic data (age, gender, marital status, socio-economic level), personal and family history, disease characteristics (age of onset, date of diagnosis, number and average duration of hospitalizations), current treatment methods, the traumatic event and its characteristics. Several scales were used: PANSS (to assess the severity of the illness), Insight Test Q8: to assess the patient's insight, MINI validated in Arabic [17]: to look for the diagnosis of PTSD and suicidal ideation and the Calgary scale validated in Arabic [18]: to look for depression.

All variables were summarized using descriptive statistics. Categorical variables were described in terms of proportions, and quantitative variables were described in terms of mean, extreme values and standard deviation. Furthermore, univariate analysis was performed; the CHI2 test was used to compare the percentage and a Student's test was conducted to compare the means. A p < 0.05 was considered significant, and the results were expressed as the odds ratio (OR) and its 95% confidence interval (95% CI). Statistical analysis was performed using SPSS version 21.0 (Statistical Package for Social Sciences Version 21 for Windows). This study has been reported in line with the STROBE Guidelines [19].

## 3 Results

### 3.1 Descriptive results

#### 3.1.1 Sample

The number of patients included in our study was 187, of whom 145 patients (78%) were seen as outpatients and 42 patients (22%) as inpatients.

#### 3.1.2 Socio-demographic characteristics

The mean age of the patients was 41.45 ± 13.273 years, with a minimum of 18 years and a maximum of 88 years. The majority of patients were male with a percentage of 76% (N = 143). Most of our patients are single with a percentage of 62% (N = 116). Only 21 patients (11%) had a regular professional activity. Only 22 patients (12%) had a university degree. Twenty patients (11%) had a secondary education. While 33 of our patients (17%) have never been to school. 88% (N = 163) of the subjects are of low socio-economic level.

#### 3.1.3 Antecedents

At least one medical or surgical history was found in 48 patients. Tobacco is the most consumed substance. It is used in 53% of patients, followed by cannabis in 29%, then alcohol in 13% of cases. A significant percentage of patients (22%) had at least one judicial record and 17% of patients had made at least one suicide attempt. Among the 187 cases, 100 patients reported the notion of one or more family antecedents, which were dominated by psychiatric antecedents (67%).

#### 3.1.4 Data on schizophrenia

The average age of schizophrenia's onset was 26.64 ± 9.82 years, with extremes ranging from 14 to 80 years. The mean duration of the disease was 173.78 months ± 125.25 with extremes ranging from 1 month to 576 months. In addition to this, the mean PANSS score of all patients is about 41.74 ± 17.55; the mean PANSS positive symptom score (PANSS P) is 11.78 ± 6.54; the mean PANSS negative symptom score (PANSS N) is 8.90 ± 4.05. The percentage of patients who were hospitalized at least once was 76% while the average number of hospitalizations was 2 ± 2.20 with extremes ranging from 0 to 15 times.

#### 3.1.5 Therapeutic treatment

Classic neuroleptics represent 55% of all prescriptions while antidepressants only account for 6% of prescriptions. In our study, more than half of the patients (84.5%) comply well with their treatments and 65% of the patients have a good insight according to the Q8 insight scale.

#### 3.1.6 Stressful events

Among the 187 patients included in our study, 65 patients (35%) experienced one or more stressful events in their lives. Also, a predominance of “physical aggression” (18.57%) was noted, followed by “witnessing violence or death” (17.27%), then “sexual abuse” and “traffic accident” (11.17%) for each of the last two stressful events. The majority of patients (81%) experienced stressful events prior to schizophrenia.

#### 3.1.7 PTSD

Among the 65 patients who were exposed to psycho-traumatic events, 25 patients have a current post-traumatic stress disorder; therefore, the prevalence of post-traumatic stress disorder in our sample is e 14%.

#### 3.1.8 Comorbidities

In our study, 27 patients (15%) were at risk for suicide, rated as “mild” to “severe” on the MINI scale. However, 56 patients (or 30%) have depression according to the CALGARY scale.

### 3.2 Analytical results

We analyzed and assessed the possible correlations between comorbidity and socio-demographic data, different histories, stressful events and distinct clinical data as well as different treatments taken by our patients. In this sense, we divided our sample into two groups:

- Group 1: Patients with current PTSD (26 cases).
- Group 2: Patients without current PTSD (161 cases).

The following Table 1, Table 2, Table 3, Table 4, Table 5 show respectively the possible correlations between the studied comorbidity and the different socio-demographic data, the history, the stressful events, the clinical data and the different treatments.

**Table 1:** Possible correlations between the comorbidity (Schizophrenia-PTSD) and socio-demographic data.

The variables		Absence of current PTSD N = 161	Presence of current PTSD N = 26	P
Age	<25 years old	12 (7,5%)	4 (15,5%)	0,906
	25–50years old	112 (69,5%)	16 (61,5%)	
	>50 years old	37 (23%)	6 (23%)	
Gender	Man	126 (78,3%)	17 (65,4%)	0,210
	Woman	35 (21,7%)	9 (34,6%)	
Origin	Urban	134 (83,3%)	20 (77%)	0,415
	Rural	27 (16,7%)	6 (23%)	
Marital status	Single	104 (64,6%)	12 (46,2%)	0,235
	Married	36 (22,3%)	8 (30,7%)	
	Widowed	4 (2,5%)	2 (7,7%)	
	Divorced	17 (10,6%)	4 (15,4%)	
Educational level	High school level or less	27 (16,8%)	6 (23%)	0,345
	More than secondary school	134 (83,2%)	20 (77%)	
Monthly revenue	<2000DH	139 (86,3%)	24 (92,3%)	0,609
	2000-5000DH	18 (11,2%)	2 (7,7%)	
	>5000DH	4 (2.5%)	0 (0%)

**Table 2:** Possible correlations between the comorbidity and different antecedents.

The variables		Absence of current PTSD N = 161	Presence of current PTSD N = 26	P
Medical/surgical history	Absence	122 (75,7%)	19 (73%)	0,767
	At least one	39 (24,3%)	7 (27%)	
Use of substances	No	62 (38,5%)	14 (53,8%)	0,140
	Yes	99 (61,5%)	12 (46,2%)	
Judicial history	No	123 (76,4%)	23 (88,4%)	0,168
	Yes	38 (23,6%)	3 (11,6%)	
Suicidal history	No	136 (72,8%)	20 (77%)	0,337
	Yes	25 (13,4%)	6 (23%)	
Familial pathological history	No	75 (46,6%)	12 (53,9%)	0,967
	Yes	86 (53,4%)	14 (46,1%)

**Table 3:** Possible correlations between the comorbidity and stressful events.

The variables		Absence of current PTSD N = 161	Presence of current PTSD N = 26	P
Stressful events	No	122 (75,7%)	0 (0%)	0,000*
	Yes	39 (24,3%)	26 (100%)	
Stressful events/schizophrenia	Before schizophrenia	10 (6,2%)	3 (11,6%)	0,195
	After schizophrenia	31 (19,2%)	23 (88,4%)

**Table 4:** Possible correlations between comorbidity and different clinical data.

The variables		Absence of current PTSD N = 161	Presence of current PTSD N = 26	P
Age of onset of schizophrenia	<20 years old	36 (22,4%)	5 (19,3%)	0,069
	>20years old	125 (77,6%)	21 (80,7%)	
Duration of schizophrenia	<155 months	79 (49%)	16 (61,5%)	0,124
	>155 months	82 (51%)	10 (38,5%)	
Number of hospitalizations	Never	33 (20,5%)	12 (46,2%)	0,544
	At least once	128 (79,5%)	14 (53,8%)	
PANSS P	<10	96 (59,6%)	6 (23%)	0,031*
	10–20	50 (31%)	14 (54%)	
	>20	15 (9,4%)	6 (23%)	
PANSS N	<10	124 (77%)	14 (53,8%)	0,005*
	>10	37 (23%)	12 (46,2%)	
PANSS G	<20	101 (62,7%)	8 (30,7%)	0,021*
	20–40	51 (31,7%)	17 (65,5%)	
	>40	9 (5,6%)	1 (3,8%)	
SSuicide risk	Absent	146 (90,7%)	14 (53,8%)	0,000*
	Mild	10 (6,2%)	8 (30,7%)	
	Medium	2 (1,2%)	3 (11,6%)	
	Severe	3 (1,9%)	1 (3,8%)	
Depression	No	119 (74%)	12 (46,2%)	0,004*
	Yes	42 (26%)	14 (53,8%)	
Insight	Bad	39 (24,2%)	5 (19,3%)	0,578
	Good	122 (75,8%)	21 (80,7%)

**Table 5:** Possible correlations between comorbidity and different treatments.

The variables		Absence of current PTSD N = 161	Presence of current PTSD N = 26	P
Compliance	Bad	25 (15,5%)	4 (15,4%)	0,985
	Good	136 (84,5%)	22 (84,6%)	
Neuroleptics	Only one	21 (13%)	4 (15,4%)	0,757
	Two or more	140 (87%)	22 (84,5%)	
Antidepressants	No	138 (85,7%)	20 (77%)	0,250
	Yes	23 (14,3%)	6 (23%)	
Benzodiazepines	No	148 (92%)	22 (84,6%)	0,264
	Yes	13 (8%)	4 (15,4%)	
Trihexyphenidyl	No	76 (47,2%)	12 (46,2%)	0,546
	Yes	85 (52,8%)	14 (53,8%)	

## 4 Discussion

Exposure to traumatic events is a very substantial component in patients with schizophrenia. A study of 275 subjects with severe mental disorders (including 64 subjects with schizophrenia) found that 98% of the cases had been exposed, at least once in their lives, to a traumatic event [4]. Another study by Piken and al. showed that 91% of patients with schizophrenia associated with substance use disorders had experienced at least one stressful event in their lives [11]. This is while Alvarez et al. in a study conducted on 102 patients of which 51% patients with schizophrenia to determine the rate of post-traumatic stress disorder, exposure to stressful events was noted in 47.5% of cases [20]. The rate of exposure to traumatic events in our sample is lower than the literature's data including schizophrenia among other psychiatric diagnoses (Table 6).

**Table 6:** Percentage of exposure to traumatic events and post-traumatic stress disorder in each study and the scales used.

Author	Number of patients	Scales	Percentage of exposure to traumatic events	Percentage of PTSD in patients with schizophrenia
Seow et al., 2016	Literature review: 34 articles	SCID, MINI, PDS, CAPS, PCL	–	0 à 57%
Achim et al., 2011	Literature review: 52 articles	SCID	–	12,4%
Mueser et al., 1998	275 patients (64 SCHZ)	PCL	98%	43%
Neria et al., 2002	426 patients (38,7% SCHZ) 24 mois 1er Episode psychotique	Questionnaire	–	14,3%
Picken et al., 2011	110 patients (79%) SCHZ + TUS	PDS, CAPS	91%	28%
Alvarez et al., 2012	102 (51% SCHZ)	Traumatic Life Events Questionnaire, Distressing Event Questionnaire	47,5%	15,1%
Our series	187 (100%SCHZ)	MINI PANSS	35%	14%

CAPS: Clinician Administered Posttraumatic stress disorder Scale; PTSD: Posttraumatic stress disorder; MINI: Mini International Neuropsychiatric Interview; PDS: Posttraumatic Diagnostic Scale; PCL: PTSD Checklist; SCID: Structured Clinical Interview for DSM; TS: Suicide attempt; TUS: Substance use disorder; SCHZ: Schizophrenia.

The prevalence of PTSD varies from one study to another. It was 43% in the study by Mueser et al. [18], 28% in the study by Picken et al. [11] and 15% in the study by Alvarez et al., [20]. In a study which evaluated 426 patients after a hospitalization of 24 months due to a first psychotic episode [5]. This cohort included 38.7% of patients with a diagnosis of schizoaffective disorder or schizophrenia. Of the total cohort, 68.5% had an exposure to a traumatic event (DSM 3-R definition). The prevalence of PTSD in the entire cohort was 14.3%. This result is very similar to that of our own study (Table 6).

The distinctions in PTSD spread rates reported in the literature could be attributed to the differences in the scales used, as well as the samples and the population targeted by the studies and the related socio-demographic data.

Young adults seem to be the most affected by the schizophrenia-post-traumatic stress disorder association [5,21,22]. The results of our study tend to go along with the data cited above, which could be due to the frequency of stressful events at this age or during childhood that manifested themselves later. A female predominance in the schizophrenia-ESPT association has been objectified by the majority of studies interested in this association [5,9,23].

It is common in the literature that patients with schizophrenia or schizoaffective disorder tend to engage in material use. PTSD symptoms are higher and more severe in substance use disorder associated with schizophrenia or schizoaffective disorder according to a comparative study of 122 subjects with and without a traumatic history [6]. In a 2001 study that dealt with 172 patients suffering from schizophrenia or schizoaffective disorder, the presence of a judicial history in patients with PTSD was about 48% [24].

Three groups of veteran patients were the subject of a study conducted by Sautter et al. [10] in 1999. In fact, they highlighted more violent and frequent behaviors when psychotic disorder is associated with PTSD, which could lead to frequent incarceration. The same study [10], found higher scores of general and psychotic positive symptoms on the PANSS and more violent thoughts and behaviors in the group with the association of PTSD and a psychotic disorder. Concerning the correlation between the severity of positive symptoms and the presence of PTSD, the results are contradictory [7]. Some authors have found an increase in positive psychotic symptoms when schizophrenia and PTSD are combined [11,14,25]. On the other hand, in other studies, no association between the severity of PTSD and the severity of positive psychotic symptoms in schizophrenia have been found [9,13,[26], [27], [28]]. However, Resnik et al. noted greater emotional distress (depressive symptomatology, anxiety, guilt, active social avoidance) when the association of PTSD with schizophrenia or schizophrenia spectrum disorder was reported [8].

While Steel et al. investigated 110 patients suffering from a psychotic disorder (schizophrenia, schizoaffective disorder or schizophreniform disorder) for the characteristics of acoustic-verbal hallucinations according to the presence or absence of a post-traumatic stress disorder, they found no difference in terms of frequency of hallucinations between schizophrenia with or without PTSD. Nevertheless, hallucinations were experienced as more intense and distressing in subjects with PTSD [27]. In our series, positive symptoms of schizophrenia were significantly correlated with the presence of PTSD.

Strauss et al., suggested to study the link amid post-traumatic stress disorder and negative symptomatology in 70 subjects with schizophrenia [12]. Two hypotheses were put forward. The first is that patients with primary negative symptoms (intrinsic to the illness) have a lower probability of developing PTSD when confronted with a traumatic event. These patients with primary negative symptoms experienced less negative emotions related to the development of PTSD (irritability, hostility, sadness, etc.). The second hypothesis would be the increase in secondary negative symptoms during the development of a post-traumatic stress state in individuals suffering from schizophrenia without primary negative symptoms. The results validated both hypotheses. More specifically, Harrison and Fowler [29] studied the link between negative symptoms and traumatic reactions in relation to the psychotic disorder or hospitalization in psychiatry. The authors were also interested in the possible link between traumatic reactions and autobiographical memory. Moreover, the results of the study suggest that people who avoid traumatic memories related to psychotic disorder and hospitalization would have more negative symptoms and less recovery of specific autobiographical memories. In our study, the severity of negative symptoms was significantly related to the presence of PTSD.

There was also a significant correlation between depression and the presence of PTSD. This corroborates the data in the literature [13,14,30]. A study carried out by Duke et al. claimed that about 94 patients showed an increase in depressive symptoms when schizophrenia was associated with PTSD [14]. Another study on the impact of post-traumatic stress disorder in subjects with schizophrenia showed a significant worsening of depressive symptoms in the presence of post-traumatic stress disorder [13].

Many studies have been interested in investigating suicidal behavior in patients with schizophrenia disorder and posttraumatic stress disorder [15,31]. A study of 165 war veterans with schizophrenia or schizoaffective disorder found a significant association between the presence of PTSD and suicidal ideation in these patients. Self-aggressive behaviors and a history of suicide attempts were more common when PTSD was associated [31]. Tarrier and Picken conducted a study in 110 subjects with a psychotic disorder (79% schizophrenia, 12% schizoaffective disorder, 1% schizophreniform disorder, 8% undifferentiated schizophrenia) associated with a substance use disorder. They found that 63% of the subjects had made at least one suicide attempt in the past, 35% reported current suicidal ideation, 21% with a suicidal scenario. Suicidal behaviors were higher with increased exposure to traumatic events and more frequent in individuals with associated post-traumatic stress disorder. Loss of hope has remained a mediator to partially explain the association between PTSD and suicidal behavior [15]. It is a frequent symptom in patients suffering from schizophrenia and is thought to be increased by PTSD. The traumatic experience would lead to cognitive alterations with an impression of a blocked future, of a hostile and threatening environment. The interpretative tendencies present in schizophrenia would potentiate this feeling of threat and would privilege social isolation, limiting access to care. Hyper-vigilance would also play a role in the development of these suicidal behaviors as well as the irritability frequently found in post-traumatic stress disorder. Substance use disorders frequently associated with schizophrenia and PTSD increase the loss of hope and make the treatment more complex [15].

In our series, we note that patients with the studied comorbidity have significantly higher suicidal risk than patients without current PTSD, which is in favor of the results described in the literature. This could be exclusively related to the presence of PTSD, as it could be related to the existence of depression as another comorbid disorder.

Calhoun et al. [16] have shown that schizophrenia and PTSD comorbidity increases somatic care utilization and the number of psychiatric hospitalizations. Nonetheless, in our study, an association between this comorbidity and data related to hospitalization was not observed. This result may be related to the fact that the majority of patients in our study were seen on an outpatient basis, which reflects a stability of symptoms and a lower hospitalization rate.

The results of our study underline a non-significant difference in terms of prescription of anti-depressants in patients with the comorbidity. However, this is the reference treatment in the presence of this comorbidity. This could be justified by the non-diagnosis of PTSD and indeed a poor management of these patients. On the other hand, no difference was found during the comparison which was made amid the two groups of patients with and without PTSD in terms of neuroleptic treatment. They took more conventional neuroleptics than atypical neuroleptics because the socio-economic level and monthly income of the patients were low.

None of our patients received cognitive-behavioral therapy or EMDR (Eye Movement Desensitization and Reprocessing), despite the fact that these techniques have been shown to be effective among patients with this comorbidity [[32], [33], [34], [35]]. This thus could be related to the lack of health professionals trained in these therapies.

## 5 Conclusion

The comorbidity schizophrenia-PTSD exists with unavoidable prevalence. It is often under-diagnosed and little sought after in current clinical practice fearing the exacerbation of psychotic symptoms or because of the existing few knowledge of the possibilities of treating the symptoms once detected and found.

The association of post-traumatic stress disorder with schizophrenia has an essential impact on the positive and the negative symptoms of schizophrenia. It could be responsible for the onset of depression and the increase of suicide's risks.

The risks of non-diagnosis of this comorbidity could lead to inappropriate treatments, a multiplication of care with no notable clinical improvement, poor therapeutic compliance and an alteration in patients’ quality of life. Therefore, there is a need for screening using known scales in order to obtain an accurate diagnosis and to highlight other disorders if they exist, or at least to minimize the risk of erroneous diagnosis.

## Sources of funding

None.

## Ethical approval

The research was carried out under the guise of anonymity. It does not concern any personal data that could directly or indirectly identify a specific person.

## Consent

Verbal informed consent was obtained from the patient for publication of this manuscript.

## Author contribution

Salah-Eddine El Jabiry: Study concept, Data collection, Data analysis, Writing the article. Mohamed Barrimi: Supervision and data validation. Bouchra oneib: Supervision and data validation. Fatima El Ghazouani: Supervision and data validation.

## Registration of research studies

**Research Registry Unique Identifying Number:** researchregistry7542.

Hyperlink: https://www.researchregistry.com/browse-the-registry#home/registrationdetails/61e0578a48c91e001e357082/

## Guarantor

Salah-Eddine El Jabiry.

## Declaration of competing interest

The authors declare no competing interest.
